# Predictive modeling of proliferative vitreoretinopathy using automated machine learning by ophthalmologists without coding experience

**DOI:** 10.1038/s41598-020-76665-3

**Published:** 2020-11-11

**Authors:** Fares Antaki, Ghofril Kahwati, Julia Sebag, Razek Georges Coussa, Anthony Fanous, Renaud Duval, Mikael Sebag

**Affiliations:** 1 Department of Ophthalmology, Université de Montréal, Montreal, QC Canada; 2 Department of Ophthalmology, Centre Hospitalier de l’Université de Montréal (CHUM), Montreal, QC Canada; 3 Centre Universitaire d’Ophtalmologie (CUO), Hôpital Maisonneuve-Rosemont, CIUSSS de l’Est-de-l’Île-de-Montréal, Montreal, QC Canada; 4 Institut National des Sciences Appliquées de Toulouse (INSA Toulouse), Toulouse, France; 5 École de Technologie Supérieure (ÉTS), Montreal, QC Canada; 6 Department of Ophthalmology and Visual Sciences, Carver College of Medicine, University of Iowa, Iowa City, IA USA; 7 Faculty of Medicine, McGill University, Montreal, QC Canada

**Keywords:** Predictive markers, Risk factors, Outcomes research

## Abstract

We aimed to assess the feasibility of machine learning (ML) algorithm design to predict proliferative vitreoretinopathy (PVR) by ophthalmologists without coding experience using automated ML (AutoML). The study was a retrospective cohort study of 506 eyes who underwent pars plana vitrectomy for rhegmatogenous retinal detachment (RRD) by a single surgeon at a tertiary-care hospital between 2012 and 2019. Two ophthalmologists without coding experience used an interactive application in MATLAB to build and evaluate ML algorithms for the prediction of postoperative PVR using clinical data from the electronic health records. The clinical features associated with postoperative PVR were determined by univariate feature selection. The area under the curve (AUC) for predicting postoperative PVR was better for models that included pre-existing PVR as an input. The quadratic support vector machine (SVM) model built using all selected clinical features had an AUC of 0.90, a sensitivity of 63.0%, and a specificity of 97.8%. An optimized Naïve Bayes algorithm that did not include pre-existing PVR as an input feature had an AUC of 0.81, a sensitivity of 54.3%, and a specificity of 92.4%. In conclusion, the development of ML models for the prediction of PVR by ophthalmologists without coding experience is feasible. Input from a data scientist might still be needed to tackle class imbalance—a common challenge in ML classification using real-world clinical data.

## Introduction

Despite advances in retinal detachment surgery, proliferative vitreoretinopathy (PVR) remains an important barrier for long-term successful anatomic repair of a rhegmatogenous retinal detachment (RRD)^1,2^. PVR has a cumulative risk of 5–10% of all retinal detachment repairs and is responsible for 75% of all primary surgical failures^1^. Several clinical and biological risk factors have been identified in the past two decades for PVR formation including trauma, aphakia, vitreous hemorrhage, and pre-existing PVR^3–9^.

Multiple formulas have been developed to predict PVR based on genetic and clinical variables but their poor predictive performance made them unsuitable for routine clinical use^10–13^. Ideal predictive formulas should identify high-risk cases in order to guide future clinical management^14^. While artificial intelligence (AI) solutions have been broadly applied to imaging data in ophthalmology, a limited number of studies have utilized AI techniques with clinical data obtained from electronic health records (EHRs)^15^. In the case of PVR, part of the difficulty of building predictive models using clinical data lies in the low incidence of the disease within the clinical cohorts, leading to imbalanced datasets^15,16^.

As of recently, the development of machine learning (ML) predictive models in healthcare had been reserved for AI experts with knowledge of coding and computer science. The feasibility of deep-learning design (a subset of AI) by physicians without coding experience was demonstrated in a first-of-its-kind report by Faes et al. in 2019^17^. This major advance in the democratization of AI was made possible by the release of automated ML (AutoML) programs by major companies allowing any individual to develop high-quality AI models^18^.

In this study, two ophthalmologists without coding experience built ML predictive classification models using an interactive application in MATLAB (MathWorks, Natick, MA) and explored the discriminative performance of those models for the prediction of postoperative PVR. To our knowledge, this is the first study examining the feasibility of AutoML prediction of postoperative complications of vitreoretinal surgery.

## Methods

The study included consecutive patients who underwent RRD repair using pars plana vitrectomy by a single surgeon (MS) from 2012 to 2019, at the Centre Hospitalier de l’Université de Montréal (CHUM), a tertiary teaching hospital in Montreal, Quebec, Canada. The institutional review board approved the study (CÉR CHUM: 19.227) and granted a waiver for informed consent given the retrospective and anonymous nature of the study. The study adhered to the tenets of the Declaration of Helsinki and was compliant with our hospital guidelines regarding the clinical data collection and extraction from EHRs.

The primary outcome of this study was the discriminative performance of ML models, developed using AutoML by ophthalmologists without coding experience, in correctly predicting PVR. Prediction of PVR was examined by area under the receiver operatic characteristic curve (AUC). Sensitivity, specificity, and positive and negative predictive values (PPV and NPV) of the ML models are the secondary outcome measures.

### Study cohort

We included simple RRD as well as those associated with giant retinal tears, vitreous hemorrhage, and pre-existing PVR grade C and/or worse (based on the updated version of the Retina Society Classification)^19^. Patients with a history of previous ocular surgery such as failed pneumatic retinopexy, prior scleral buckling, vitrectomy for a non-RRD indication, glaucoma, and cataract surgery were also eligible for inclusion in the study. We excluded patients who were younger than 18 years, those with tractional retinal detachments, recent penetrating trauma (< 6 months), and those who have previously undergone pars plana vitrectomy for RRD in the same eye. All cases were reviewed by a research fellow (JS) and an experienced vitreoretinal surgeon (MS) before inclusion in the study.

### Data set preparation and feature collection from the EHR

We collected 15 features from the EHR as described in Table 1. All categorical features were evaluated based on the presence or absence of the clinical examination finding at the preoperative clinical encounter, with the exception of “postoperative lens status” that was evaluated on postoperative day 1. For this feature, pseudophakia and aphakia were counted together. Pre-existing PVR was defined as the presence of PVR Grade C involving more than 1 clock hour of the retina, according to the updated version of the Retina Society Classification^19^. Although we collected intraoperative parameters such as type of endotamponade and use of endolaser and cryotherapy, we elected to exclude those surgeon factors from the predictive models in order to limit confounding.

**Table 1:** Collected clinical variables from the EHR.

Category	Variable
Sociodemographic characteristics	Age in years [continuous], sex [binary]
Past ocular history	Previous ocular surgery [binary]
History of present illness	Duration of symptoms in days [continuous]
Retinal detachment characteristics	Subtotal/ total retinal detachment (≥ 3 quadrants) [binary], macular status [binary], pre-existing PVR [binary], vitreous hemorrhage [binary], number of retinal breaks [continuous], giant retinal tear [binary]
Other examination findings	Macular hole [binary], anterior uveitis [binary], number of quadrants of lattice degeneration [polynomial], intraocular pressure in mmHg using Goldmann applanation [continuous], postoperative lens status [binary]

The variables included sociodemographic and past ocular history data as well as retinal detachment characteristic and other relevant examination findings.

*EHR *electronic health records, *PVR *proliferative vitreoretinopathy.

### Classification procedure

The outcome of interest was development of postoperative PVR in patients with at least 3 months of follow-up. Patients who developed PVR during follow-up were labeled as “PVR” and those who did not develop PVR during follow-up were labeled as “No PVR.” The endpoint definition was based on a landmark study in the field by Kon et al.^11^. Postoperative PVR was defined as either the presence of new PVR Grade C involving more than 1 clock hour in the detached retina after vitrectomy or new clinically visible membranes or bands of greater than 1 clock hour in a successfully attached retina. Any new visible macular pucker that was visually significant and required surgical re-intervention was also included in the PVR group. The presence of absence of macular pucker was determined clinically by dilated fundus examination. The labeling was performed by an ophthalmologist-in-training (FA) and an experienced vitreoretinal surgeon (MS) based on the retrospective review of the data written in the EHR. The EHR data included examination findings during the postoperative period as well as operative reports from subsequent surgeries.

### Data set preparation before model development

**Figure 1:**
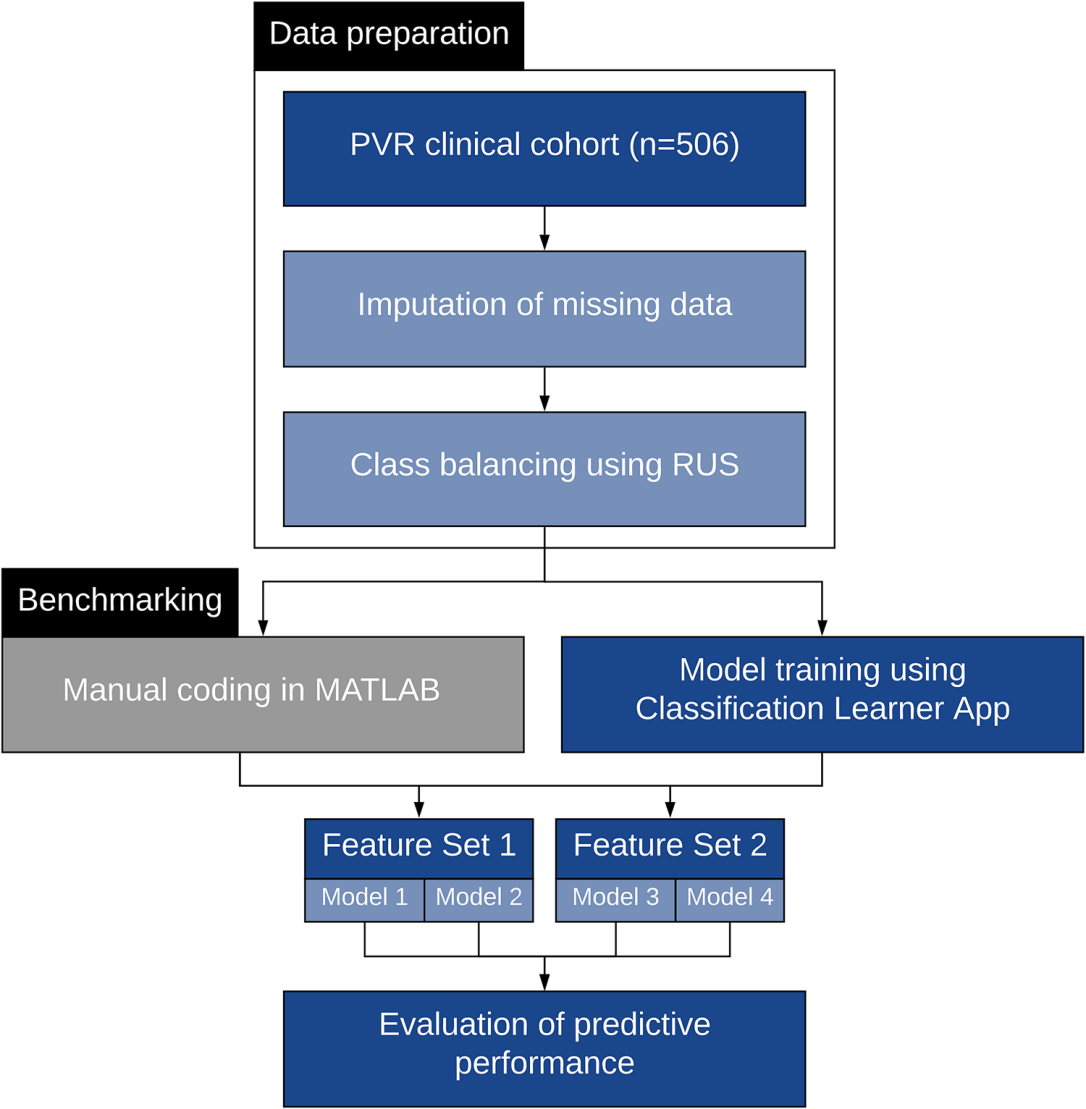
This diagram demonstrates the workflow for predictive modeling of proliferative vitreoretinopathy (PVR) using automated machine learning (AutoML). After data preparation and class balancing using random undersampling (RUS), the two ophthalmologists used the Classification Learner App to design support vector machine (SVM) and Naïve Bayes (NB) algorithms. Details about the feature sets and models are described in the methodology. In parallel to the design by the two ophthalmologists, manually coded algorithms were also prepared as a benchmarking measure.

A scientist with knowledge in data mining (GK) prepared the dataset. Figure 1 summarizes the dataflow during the study. Within the total clinical cohort of 506 eyes, 460 were No PVR (majority class) and 46 were PVR cases (minority class of interest), representing a 9.1% incidence of PVR. Complete data were available for all categorical variables. For continuous variables, 77 data points (15.2%) were missing for the “duration of symptoms” variable and 90 data points (17.8%) were missing for the “intraocular pressure” variable. We conducted a missing value analysis and found the data to be Missing Completely at Random (MCAR) using Little’s MCAR test (p = 0.933). Thus, imputation using the median of the non-missing values in the variable column was performed.

Before the dataset could be used for training by the two ophthalmologists (FA and RGC), we employed data-level solutions to tackle the imbalanced dataset problem. This was important to perform prior to any data training in order to not undermine the predictive performance of the models trained by the two ophthalmologists. We performed random undersampling (RUS) of the majority class using Waikato Environment for Knowledge Analysis (WEKA), an open-source data-mining software made available by the University of Waikato in New Zealand^20^. We set the case:control ratio to 2:1, leading to a new dataset containing 92 controls and 46 PVR cases and a disease prevalence of 33.33%. While higher matching ratios could have been used, we used this ratio to avoid perpetuating the class imbalance problem^21^. The clinical characteristics of the original and new datasets are presented in Table 2 and Supplementary Table S1, respectively. The significantly different features remained unchanged in the new dataset, highlighting the absence of significant change in the distribution of the data.

**Table 2:** Clinical characteristics of patients in the PVR and no PVR groups.

	PVR (n = 46)	No PVR (n = 460)	P value
**Age, years**			< 0.001
Mean	68.78	59.93	
Standard deviation	8.448	11.931	
Median	68.50	61.00	
Range	48–90	18–95	
**Sex**			0.743
Male	30 (65.2%)	311 (67.6%)	
Female	16 (34.8%)	149 (32.4%)	
**Previous surgery**			0.650
Yes	7 (15.2%)	60 (13.0%)	
No	39 (84.8%)	400 (87.7%)	
**Duration of symptoms, days**			< 0.001
Mean	35.05	16.73	
Standard deviation	62.429	39.598	
Median	21.00	7.00	
Range	1–365	0–365	
**Subtotal/total RRD**			< 0.001
Yes	22 (47.8%)	59 (12.8%)	
No	24 (52.2%)	401 (87.2%)	
**Macular status**			0.001
On	8 (17.4%)	193 (42.0%)	
Off	38 (82.6%)	267 (58.0%)	
**Pre-existing PVR**			< 0.001
Yes	17 (37.0%)	1 (0.2%)	
No	29 (63.0%)	459 (99.8%)	
**Vitreous hemorrhage**			0.002
Yes	10 (21.7%)	31 (6.7%)	
No	36 (78.3%)	429 (93.3%)	
**Number of tears**			0.323
Mean	2.41	2.03	
Standard deviation	2.296	1.569	
Median	2.00	1.00	
Range	1–10	0–9	
**Giant tear**			0.023
Yes	4 (8.7%)	9 (2.0%)	
No	42 (91.3%)	451 (98.0%)	
**Macular hole**			0.714
Yes	1 (2.2%)	23 (5.0%)	
No	45 (97.8%)	437 (95.0%)	
**Uveitis**			0.006
Yes	3 (6.5%)	2 (0.4%)	
No	43 (93.5%)	458 (99.6%)	
**Quadrants of lattice degeneration**			0.441
0	31 (67.4%)	301 (65.4%)	
1	12 (26.1%)	91 (19.8%)	
2	1 (2.2%)	46 (10.0%)	
3	1 (2.2%)	14 (3.0%)	
4	1 (2.2%)	8 (1.7%)	
**Intraocular pressure**			0.002
Mean	11.40	14.69	
Standard deviation	5.065	5.039	
Median	12.00	15.00	
Range	0–20	0–60	
**Postoperative lens**			0.759
Phakic	25 (54.3%)	237 (51.5%)	
Pseudophakic/aphakic	21 (45.7%)	223 (48.5%)	

The comparison between the “PVR” and “No PVR” groups was performed using Mann–Whitney U test for all continuous variables. For categorical variables, we used Chi-square and Fisher’s exact tests (for cells with expected counts < 5).

*PVR *proliferative vitreoretinopathy, *RRD *rhegmatogenous retinal detachment.

### Predictive model building and feature selection

As shown in Supplementary Video 1, two ophthalmologists (FA and RGC) with no previous coding experience performed the model development after a period of self-study. The ophthalmologists were asked to consult the “Classification Learner App” documentation in the help center provided by MathWorks (https://www.mathworks.com). The documentation included illustrated step-by-step articles on how to train the models and explore the reported diagnostic measures. The ophthalmologists were asked to train all classifiers, compare predictive performances, and choose the two best models that worked for the classification problem for each set of features. Model tuning was performed using the built-in AutoML feature to find the best hyperparameters to optimize performance. All steps were performed jointly and the ophthalmologists were allowed to revisit the documentation at any time.

The Classification Learner App allowed for easy feature selection; i.e., choosing the different clinical factors to predict PVR. To reduce overfitting and to gain a better understanding of the clinical importance of each input feature, we performed univariate feature selection on the original cohort of 506 eyes. Each clinical feature was studied individually using univariate tests, and only the features that were significantly different between the “PVR” and “No PVR” groups were included in the ML models. We used two feature sets, one that included all features found using univariate feature selection (Feature Set 1) and another that used the same variables with the exception of “pre-existing PVR” (Feature Set 2). We asked the ophthalmologists to use fivefold cross-validation (CV), a robust internal validation technique^22^. At the end, combined confusion matrices were created for performance evaluation^23^.

While we could have developed artificial neural networks and deep-learning algorithms, we elected to build binary classifiers better suited for our sample size and classification problem^24^. The two ML algorithms with the best discriminative performance were the Naïve Bayes (NB) and Support Vector Machine (SVM) classifiers. NB is a probabilistic classifier that uses the frequencies of different input features from cases in the “PVR” and “No PVR” groups during training to predict PVR when presented with a new case. SVMs expand the idea of linear classifiers and aim to find a decision boundary (hyperplane) that allows for the largest margin separation between the “PVR” and “No PVR” groups after mapping the input data to a high-dimensional space^25^.

### Model benchmarking

Because AutoML is still a relatively new technology and to corroborate the ML models obtained by the two ophthalmologists, a scientist with coding experience (GK) developed separate algorithms based on the same ML techniques found in the Classifier Learner App and performed separate analyses. This was based on the principle of redundancy in order to ensure that the predictive models obtained by the two ophthalmologists were reliable. Due to the absence of measures of variability such as confidence intervals, we could not employ statistical analyses to compare the predictive performance of the automated and manually coded models. Instead, we relied on a general “order of magnitude” comparison of the F1 score (weighted average of the precision and recall) to benchmark the discriminative performance of our automated models. The codes are available on GitHub (https://github.com/ghofok/PVR_Prediction) and their predictive performance is shown in Supplementary Table S2.

### Statistical analysis and performance metrics

Continuous variables are presented as mean, median, range, and standard deviation. Categorical variables are presented as counts and percentages. Since all the continuous variables were not normally distributed (p < 0.001, Shapiro–Wilk test), we used nonparametric tests (Mann–Whitney U). We used the two-sided Fisher’s exact test and Chi-Square test for categorical variables. We defined statistical significance as p-value < 0.05. All statistical tests were performed using SPSS v. 25.0 (SPSS, Chicago, IL). For consistency, we adhered to the standard test accuracy terminology—sensitivity (recall), specificity, PPV (or precision), and NPV—that were calculated from the confusion matrix presented in the Classification Learner App. We also calculated prevalence-adjusted PPV and NPV values to correct for the real prevalence of PVR in the overall cohort using Bayes’ theorem^26^. The AUC is also presented.

## Results

### Clinical cohort

Of the 557 eyes that met the inclusion criteria, 506 eyes were included in the final analysis. We excluded eyes with < 3 months of follow-up (n = 28) and the second operated eye for patients who had both eyes eligible at entry (n = 15). After careful case-by-case review, we excluded 8 additional eyes because we could not confidently distinguish between pre-existing PVR (input variable) and new postoperative PVR (outcome of interest) using the available EHR data. Our final cohort of 506 patients were followed for a median of 52 months (range 3–105 months), of whom 46 patients developed PVR postoperatively (9.1%). Postoperative PVR was characterized by visually significant macular pucker requiring re-intervention in 14 cases (30.4%), Grade C posterior PVR in 18 cases (39.1%) and Grade C anterior PVR in 14 cases (30.4%). Primary surgical failure occurred in 28 cases (60.9%). Anatomic success at final follow-up, defined as retinal re-attachment without long-term endotamponade with silicone oil, was seen in 33 cases (71.7%).

### Clinical risk factors for PVR

Table 2 shows the clinical characteristics of patients in the “PVR” and “No PVR” groups. Several features were significantly different between the two groups. The “PVR” group was older (p < 0.001), had a longer duration of symptoms prior to presentation (p < 0.001), and had more extensive retinal detachments characterized by subtotal/total retinal detachment (p < 0.001) and macula-off detachments (p = 0.001). The retinal detachments in the “PVR” group were more complex as characterized by the presence of giant tears (p = 0.023), vitreous hemorrhage (p = 0.002), and pre-existing PVR (p < 0.001). Lower intraocular pressure (p = 0.002) and the presence of uveitis (p = 0.006) were also more common in the “PVR” group. Based on those results, we included all statistically significant features in Feature Set 1, with the exception of “uveitis” because of its association with “intraocular pressure,” in an effort to reduce multicollinearity.

### Discriminative performance of the machine learning models

**Figure 2:**
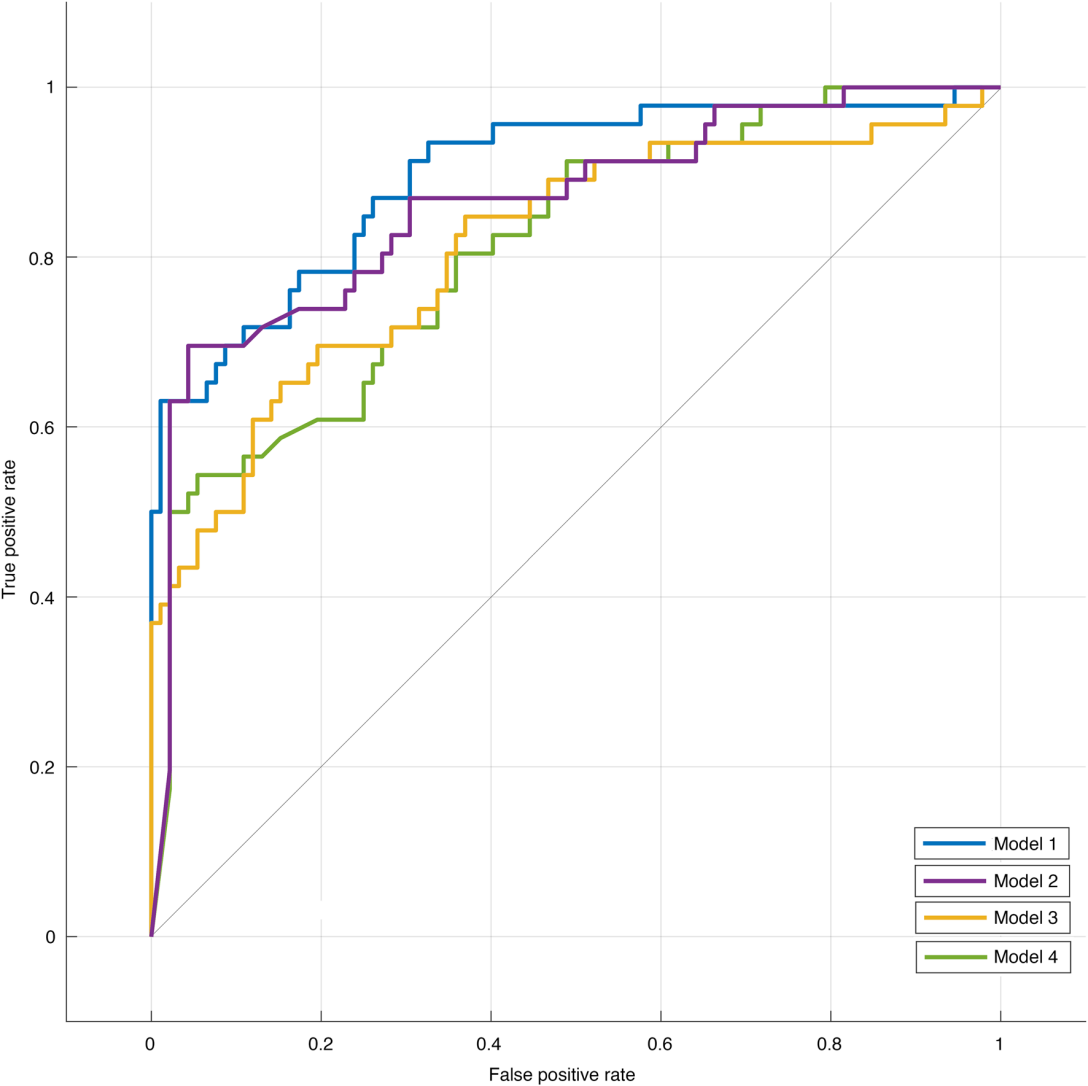
Receiver operating characteristic (ROC) curves of the discriminative performance of Models 1–4. Models 1 (quadratic Support Vector Machine [SVM]) and 2 (optimized Naïve Bayes [NB]) used Feature Set 1 that included all clinically important features. Models 3 (optimized SVM) and 4 (optimized NB) used Feature Set 2, which did not include pre-existing PVR as an input feature.

Table 3 provides a summary of the discriminative performance of all 4 ML models. The receiver operating characteristics curves (ROC) are presented in Fig. 2. The best-performing model was the quadratic SVM that included all relevant clinical variables (Model 1). The AUC was 0.90. The computed sensitivity and specificity were 63.0% and 97.8%, respectively. The PPV and NPV were 93.5% (adjusted 74.4%) and 84.1% (adjusted 96.4%) respectively. Model 2 had an AUC of 0.86, higher sensitivity (69.6%), lower specificity (95.7%), lower PPV (88.9%, adjusted 61.6%), and higher NPV (86.3%, adjusted 96.9%). Models that did not include pre-existing PVR as an input (Feature Set 2) had lower diagnostic properties. Model 3 had an AUC of 0.81. The sensitivity and specificity were 45.7% and 94.6%, respectively. The PPV and NPV were 80.8% (adjusted 45.7%) and 77.7% (adjusted 94.6%), respectively. Model 4 had an AUC of 0.81, a sensitivity of 54.3%, and a specificity of 92.4%. The PPV and NPV were 78.1% (adjusted 41.7%) and 80.2% (adjusted 95.3%), respectively.

**Table 3:** Summary of the discriminative performance of all 4 ML models.

Model	TP	FP	TN	FN	AUC	F1	SN (%)	SP (%)	PPV (%)	NPV (%)
**Feature Set 1 (8 features)**										
Model 1: Quadratic SVM	29	2	90	17	0.90	0.75	63.0	97.8	93.5	84.1
Model 2: Optimized NB	32	4	88	14	0.86	0.78	69.6	95.7	88.9	86.3
**Feature Set 2 (7 features)**										
Model 3: Optimized SVM	21	5	87	25	0.81	0.58	45.7	94.6	80.8	77.7
Model 4: Optimized NB	25	7	85	21	0.81	0.64	54.3	92.4	78.1	80.2

*ML *machine learning, *TP *true positives, *FP *false positives, *TN *true negatives, *FN *false negatives, *AUC *area under the receiver operating characteristics, *F1 *F1 score, *SN *sensitivity, *SP *specificity, *PPV *positive predictive value, *NPV *negative predictive value, *SVM *support vector machine, *NB *Naïve Bayes.

For benchmarking purposes, we compared F1 scores of the 4 automated models to the ones obtained by manual coding. As shown in Supplementary Table S2, the performances were in the same order of magnitude for all models. The F1 scores were: 0.75 (vs 0.76) for Model 1, 0.78 (vs 0.81) for Model 2, 0.58 (vs 0.60) for Model 3 and 0.64 (vs 0.69) for Model 4.

### Examples of cases

**Figure 3:**
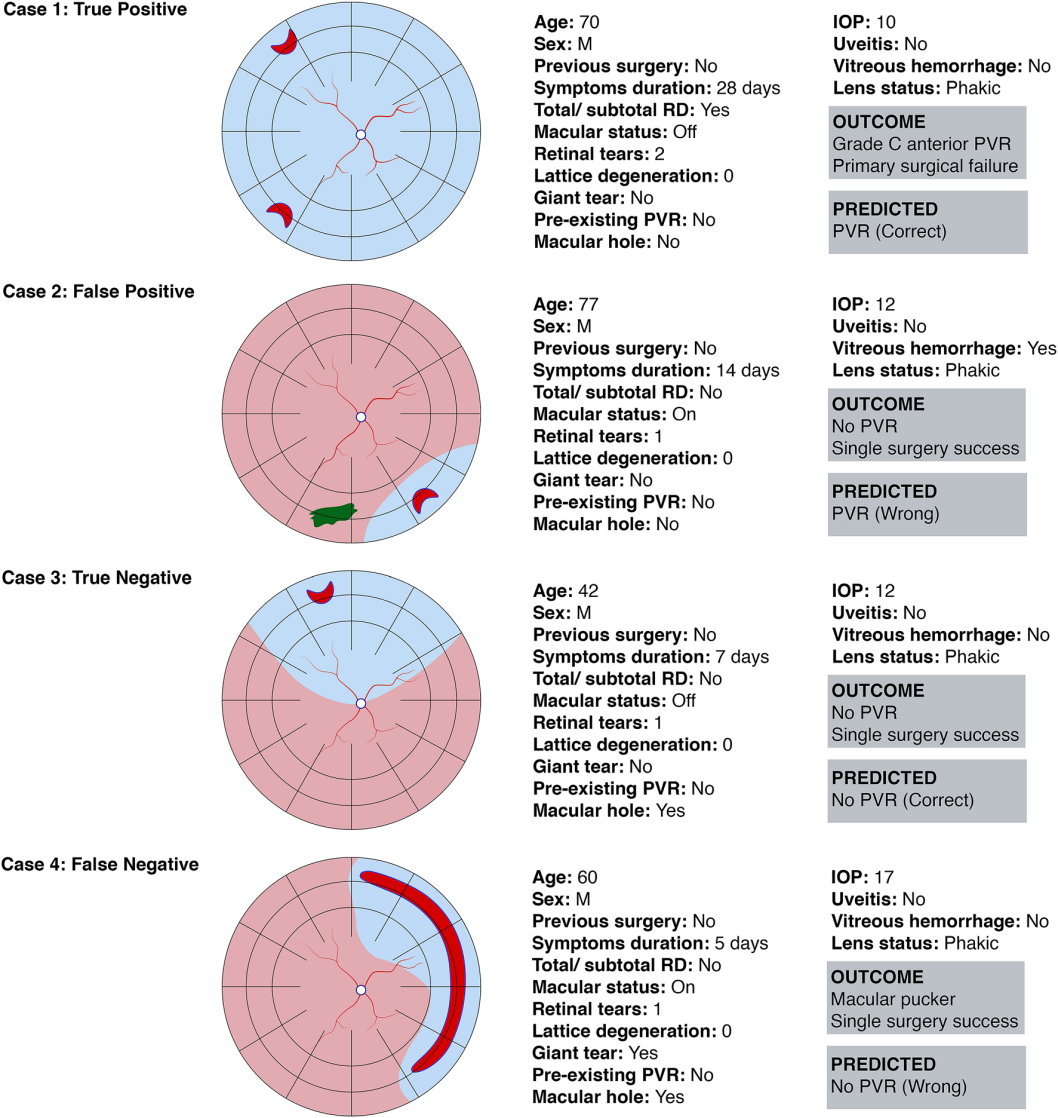
Four representative cases are shown highlighting correct classifications and misclassifications by Model 1. Case 1 illustrates a correctly classified case of PVR in an eye with a total rhegmatogenous retinal detachment (RRD) and late presentation prior to surgery. Case 3 shows a common case of simple macula-off RRD with no obvious risk factors for PVR, correctly classified as “No PVR”. In case 2, the algorithm misclassified the case as “PVR” probably due to the long duration of symptoms and the presence of vitreous hemorrhage. In case 4, the algorithm failed to predict PVR despite the presence of a giant retinal tear. The normal intraocular pressure, absence of pre-existing PVR, and macula-on status might have influenced the classifier’s decision.

Figure 3 illustrates four clinical examples from the cohort and the predicted outcome using Model 1. Cases 1 and 3 represent true positive and true negative cases correctly classified by Model 1. For case 1, it is no surprise that the long duration of symptoms coupled with the extent of the retinal detachment might be predictive of postoperative PVR. The patient developed Grade C anterior PVR and the model correctly predicted the outcome of interest. Case 3 represents a common case of simple macula-off retinal detachment associated to a single retinal tear and no obvious risk factors for PVR. The patient did not develop PVR and the model correctly predicted the outcome.

Cases 2 and 4 represent misclassified false-positive and false-negative cases. In case 2, a 77-year-old man presented with a macula-on retinal detachment with a single retinal tear and vitreous hemorrhage. The patient did not develop postoperative PVR but the model falsely predicted PVR in this case, probably due to the long duration of symptoms and the presence of vitreous hemorrhage. In case 4, a 60-year-old man presented after 5 days of symptoms with a macula-on retinal detachment associated with a giant retinal tear. He developed visually significant macular pucker that required re-intervention, but the model did not predict PVR in this case despite the presence of a giant tear. The normal intraocular pressure, absence of pre-existing PVR and macula-on status might have influenced the classifier’s decision.

## Discussion

Recent widespread adoption of EHRs has allowed the collection of large quantities of clinical data, especially in ophthalmology^15^. Data analysis from EHRs and the development of predictive models had been reserved for scientists with considerable mathematical and coding knowledge. In this study, we demonstrate that ophthalmologists without coding experience can use AutoML to develop and compare ML models to predict postoperative PVR using preoperative clinical data. Still, input from a data scientist was required for data preparation before model development could be performed. To our knowledge, this is the first report of AutoML predictive modeling of postoperative PVR following successful RRD repair.

In current clinical practice, postoperative PVR cannot be readily predicted or prevented despite strong evidence of the predisposing clinical factors for this disease. The design of predictive models for PVR may be important in facilitating clinical decision-making and supporting the research effort to find safe and effective prophylactic agents for PVR. Recent studies have suggested that future research should be conducted on high-risk patients only until a benefit is demonstrated in this subgroup^27^. As such, the identification of patients at high risk for postoperative PVR is vital to find study subjects for trials looking at prophylactic therapies. In clinical practice, the benefits of identifying those patients for counseling, to consider prophylactic treatment, and to ensure adequate follow-up cannot be overlooked.

While we could have included all 15 clinical features as inputs for the ML algorithms, we elected to perform univariate feature selection. This allowed us to select only important risk factors for PVR towards understanding the underlying process driving the predictions. Feature Set 1 included 8 features: age, duration of symptoms, intraocular pressure, extent of RRD, macular status, presence of giant tears, vitreous hemorrhage, and pre-existing PVR. Feature Set 2 included all previous features with the exception of pre-existing PVR (7 features). We intended to test Feature Set 2 because previous studies had suggested that the absence of clear distinction between preoperative and postoperative PVR might be a limiting factor for the development of models to predict PVR^4^.

Our ML models were developed as a tool for clinicians to predict the development of postoperative PVR as a basis for treatment decisions in patients with RRD who will undergo pars plana vitrectomy. As such, they should be used for diagnosis rather than screening. There was a necessary trade-off between sensitivity and specificity; while high sensitivity is always a principal objective, high specificity and PPV are more desirable in our clinical context. From our models, the algorithm with the best overall discriminative performance was Model 1—the quadratic SVM algorithm that used all selected clinical features. From a performance point of view, Model 1 showed acceptable sensitivity (63.0%) and excellent specificity (97.8%). This means that among patients who did not develop postoperative PVR, 97.8% were correctly predicted to not develop PVR (true-negative rate) and only 2.2% were falsely classified as “PVR” (false-positive rate). However, among patients who developed PVR, 63.0% were predicted to develop the complication (true-positive rate) while 37.0% were missed (false-negative rate). Given those results, our highly specific classifier should be considered helpful in ruling in PVR when the test is positive but should be interpreted with caution in the case of a negative prediction. In the undersampled cohort (33.3% PVR), the calculated PPV was 93.5% but decreased to 74% when we adjusted for a real-world prevalence of 9.1%. Practically, from a clinician’s point of view, when a patient tests positive there is 74% chance that they will develop postoperative PVR. The PPV can be higher in populations with higher prevalence of PVR, such as patients with complicated RRDs.

In the literature, one of first predictive models of PVR was described by Asaria et al., who demonstrated the feasibility of PVR prediction using clinical risk factors^10^. Kon et al. also constructed regression models to predict the probability of a patient to develop PVR^11^. The model that used clinical factors alone was reported to have “predicted the outcome in 72.8% of patients” and the model that included additional vitreous protein levels “predicted the outcome in 76.5% of patients”^11^. The use of validation measures was not reported and it remains unclear what the authors meant by “predicted the outcome” with reference to the standardized performance metrics used today. Later, Rojas et al. reported the results of the external validation of three PVR predictive formula-based linear and radial SVMs using prospective data from a multicentric study^28^. The models included clinical features in addition to genetic data. The radial SVM had 66.7% sensitivity and 81.5% specificity in one of the samples. In parallel, biomarkers from subretinal fluid obtained during vitrectomy have also been used to improve the predictive performance of PVR models^12^. In their study of 54 PVR cases with age- and sex-matched controls, Ricker et al. showed that pre-existing PVR was the only independent predictor of postoperative PVR (AUC of 0.67). When a single biomarker was included in the model, the AUC improved to 0.89^12^. Pending external validation, our automatically generated models seem to offer comparable discriminative performances.

From a methodological standpoint, our study has several strengths. Our original clinical cohort was large and included 506 eyes with a PVR rate of 9.1%. The sample size was sufficiently large to find statistically significant risk factors for PVR; however, the low incidence of PVR led to an imbalanced dataset problem, a common challenge in ML classification using real-world data^16,29^. To address this issue, data preparation was required before the two ophthalmologists could use the data for training and testing^30–32^. After exploring different resampling techniques, we found that the most suitable option was undersampling to create a case–control study design^33–35^. We used cross-validation, which is a rigorous internal validation measure that determines the robustness and generalizability of the predictive models^22^. All efforts were made to adhere to transparent reporting of a multivariable prediction model for individual prognosis or diagnosis (TRIPOD) guidelines^36^. We were able to build optimized ML models using AutoML, a feature of the software that automates and streamlines complex processes in the ML workflow such as hyperparameter tuning. This AutoML feature can significantly improve prediction accuracy^37^.

The study has some limitations. The absence of measures of variability provided by the graphical interface limited our ability to compare our models among each other and with the manually coded benchmarking models. Despite this issue, our models were generally comparable to the manually coded algorithms; however, some differences in performance were observed, possibly due to the difference in thresholds to which the algorithms were optimized. Another limitation of the study is the absence of external validation. The problem of generalizability of predictive PVR formulas has been studied in a report by Sala-Puigdollers et al.^14^. We are aware that external validation using new data is necessary before a model can be considered for use in clinical research or practice^38^.

In summary, we demonstrated the feasibility of ML model design by ophthalmologists without coding experience for the prediction of PVR using clinical data. However, it remains to be seen if clinicians with no coding experience can independently build ML models without any input from data scientists and engineers. For balanced datasets and image classification tasks, independent model building appears to be feasible. However, in the case of low-incidence diseases, clinicians will be faced with the class imbalance problem and contributions from data scientists might be indispensable. In parallel, as the technology of AutoML further matures, benchmarking with manually coded algorithms will no longer be needed. In future studies, we aim to explore the external validity of our models. In particular, we wish to study the benefits of closer postoperative follow-up for patients predicted to develop PVR and aim to look at retinal redetachment as a separate outcome. We will also be assessing the healthcare burden of unnecessarily close follow-up in false-positive cases.

## Supplementary information

Supplementary Video 1.

Supplementary Information 1.
