# Supplementary material for: Predictive modeling of proliferative vitreoretinopathy using automated machine learning by ophthalmologists without coding experience
Source: Sci Rep. 2020 Nov 11;10:19528. doi: 10.1038/s41598-020-76665-3 (PMC7658348; doi:10.1038/s41598-020-76665-3)
Supplement: Supplementary file 2 — Supplementary Information 1. [file 41598_2020_76665_MOESM2_ESM.pdf]

Predictive Modeling of Proliferative Vitreoretinopathy Using Automated Machine Learning By Ophthalmologists Without Coding Experience

Fares Antaki[1,2,3], Ghofril Kahwati[4,5], Julia Sebag[1], Razek Georges Coussa[6], Anthony Fanous[7], Renaud Duval[1,3], Mikael Sebag[1,2,*]

1. Department of Ophthalmology, Université de Montréal, Montreal, Quebec, Canada
2. Department of Ophthalmology, Centre Hospitalier de l'Université de Montréal (CHUM), Montreal, Quebec, Canada
3. Centre Universitaire d'Ophtalmologie (CUO), Hôpital Maisonneuve-Rosemont, CIUSSS de l'Est-de-l'Île-de-Montréal, Montreal, Quebec, Canada
4. Institut National des Sciences Appliquées de Toulouse (INSA Toulouse), Toulouse, France
5. École de Technologie Supérieure (ÉTS), Montreal, Quebec, Canada
6. Department of Ophthalmology and Visual Sciences, Carver College of Medicine, University of Iowa, Iowa City, Iowa, United States of America
7. Faculty of Medicine, McGill University, Montreal, Quebec, Canada

* Corresponding author. sebag.mikael@gmail.com

SUPPLEMENTARY MATERIALS

Supplementary Table S1: Clinical characteristics of patients after missing data imputation and random undersampling (RUS) of the majority class

	PVR (n = 46)	No PVR (n = 92)	P value
**Age, years**			
Mean	68.78	57.79	<0.001
Standard deviation	8.448	12.884	
Median	68.50	60.00	
Range	48 – 90	18 – 80	
**Sex**			1.000
Male	30 (65.2%)	61 (66.3%)	
Female	16 (34.8%)	31 (65.2%)	
**Previous surgery**			0.583
Yes	7 (15.2%)	10 (10.9%)	
No	39 (84.8%)	82 (89.1%)	
**Duration of symptoms, days**			<0.001
Mean	30.78	10.80	
Standard deviation	58.266	17.031	
Median	14.00	7.00	
Range	1 – 365	0 – 126	
**Subtotal/total RRD**			<0.001
Yes	22 (47.8%)	12 (13.0%)	
No	24 (52.2%)	80 (87.7%)	
**Macular status**			0.019
On	8 (17.4%)	35 (38.0%)	
Off	38 (82.6%)	57 (62.0%)	
**Pre-existing PVR**			<0.001
Yes	17 (37.0%)	0 (0.0%)	
No	29 (63.0%)	92 (100.0%)	
**Vitreous hemorrhage**			0.001
Yes	10 (21.7%)	3 (3.3%)	
No	36 (78.3%)	89 (96.7%)	
**Number of tears**			0.099
Mean	2.41	1.82	
Standard deviation	2.296	1.398	
Median	2.00	1.00	
Range	1 – 10	0 – 8	
**Giant tear**			0.042
Yes	4 (8.7%)	1 (1.1%)	
No	42 (91.3%)	91 (98.9%)	
**Macular hole**			0.424
Yes	1 (2.2%)	6 (6.5%)	
No	45 (97.8%)	86 (93.5%)	
**Uveitis**			0.035
Yes	3 (6.5%)	0 (0.0%)	

No	43 (93.5%)	92 (100.0%)	
**Quadrants of lattice**			0.338
0	31 (67.4%)	51 (55.4%)	
1	12 (26.1%)	26 (28.3%)	
2	1 (2.2%)	11 (12.0%)	
3	1 (2.2%)	3 (3.3%)	
4	1 (2.2%)	1 (1.1%)	
**Intraocular pressure**			0.008
Mean	12.26	15.58	
Standard deviation	4.669	6.708	
Median	14.00	15.00	
Range	0 – 20	9 – 60	
**Postoperative lens**			0.857
Phakic	25 (54.3%)	52 (56.5%)	
Pseudophakic/aphakic	21 (45.7%)	40 (43.5%)	

Notes: PVR = proliferative vitreoretinopathy; RRD = rhegmatogenous retinal detachment. The comparison between the “PVR” and “No PVR” groups was performed using Mann-Whitney U test for all continuous variables. For categorical variables, we used Chi-square and Fisher’s exact tests (for cells with expected counts < 5).

Supplementary Table S2: Summary of the discriminative performance of the manually coded validation models

Model	TP	FP	TN	FN	F1	SN	SP	PPV	NPV
**Feature Set 1 (8 features)**									
Manual Quadratic SVM	30	3	89	16	0.76	65.2%	96.7%	90.9%	84.8%
Manual Optimized NB	33	2	90	13	0.81	71.7%	97.8%	94.3%	87.4%
**Feature Set 2 (7 features)**									
Manual Optimized SVM	24	10	82	22	0.60	52.2%	89.1%	70.6%	78.8%
Manual Optimized NB	28	7	85	18	0.69	60.9%	92.4%	80.0%	82.5%

Notes: ML = machine learning; TP = true positives; FP = false positives; TN = true negatives; FN = false negatives; F1 = F1 score; SN = sensitivity; SP = specificity; PPV = positive predictive value; NPV = negative predictive value; SVM = support vector machine; NB = Naïve Bayes.
